# Editorial: Immune responses in the progression of allergy and asthma

**DOI:** 10.3389/fimmu.2024.1503497

**Published:** 2024-11-26

**Authors:** Ferdaus Mohd Altaf Hossain, Shaikh M. Atif, Seyyed Shamsadin Athari, Carmen Panaitescu

**Affiliations:** ^1^ Faculty of Veterinary, Animal & Biomedical Sciences, Sylhet Agricultural University, Sylhet, Bangladesh; ^2^ School of Medicine, University of Colorado Anschutz Medical Campus, Aurora, CO, United States; ^3^ Department of Immunology, Faculty of Medicine, Zanjan University of Medical Sciences, Zanjan, Iran; ^4^ Victor Babes University of Medicine and Pharmacy, Timisoara, Timis, Romania

**Keywords:** asthma, allergy, IgE, oxidative stress, epigenetic regulation

The pathogeneses of allergies and asthma are characterized by chronic airway inflammation, hyperresponsiveness, and airway remodeling, contributed to by heterogeneous, multifaceted, and complex immune mechanisms. The immune dysregulation in asthmatic cases is associated with enhanced Th2-mediated response and IgE production, which are central to disease progression. Advances in immunology have provided insights into these processes, offering new therapeutic targets. In this editorial, we will discuss the immune responses in allergy and asthma pathogenesis, focusing on the canonical Th2 cell response, IgE signaling, Regulatory CD4^+^ T cells (Tregs), oxidative stress, epigenetic regulation, and novel therapies.

The canonical allergic response is primarily mediated by the abundance of Th2 cells and the induction of pro-inflammatory cytokines, which results in eosinophilia and the class switching of B cells to IgE . IgE, in turn, binds to the high-affinity receptor FcϵRI on mast cells and basophils, sensitizing them to allergens. The subsequent allergen exposure causes cross-linking of IgE, triggering degranulation, release of histamine, and other pro-inflammatory mediators that exacerbate airway inflammation and bronchoconstriction. An elegant study by Commins et al. showed that tick bites associated with the α-Gal syndrome (AGS) resulted in an allergic response initiated by IgE overexpression against the galactose-α-1,3-galactose (α-Gal) epitope found in foods, pharmaceuticals, and many mammalian products (1).

In inflamed airways, reactive oxygen species (ROS) activate signaling pathways, like mitogen-activated protein kinases (MAPKs), which augment the inflammatory response, causing cellular influx and upregulation of matrix metalloproteinases (MMPs) and resulting in airway remodeling and chronic inflammation. A recent study by Kim et al. showed that inhibition of oxidative stress-driven pathways significantly reduces eosinophilia, pro-inflammatory cytokines, and airway inflammation, thus improving clinical outcomes in asthmatic patients.

An original research study by Wang et al. suggested that ALKBH5-mediated m6A dysregulation likely contributes to nasal inflammation through the MAPK pathway. ALKBH5-driven m6A dysregulation refers to disruptions in the normal control of m6A, a chemical modification found on RNA, caused by the enzyme ALKBH5. As an m6A demethylase, the role of ALKBH5 is to remove m6A modifications from RNA. When its function becomes dysregulated, the usual m6A modification patterns can be altered, potentially affecting gene expression, RNA stability, and other related processes. During inflammation, such imbalances might influence signaling pathways, including the MAPK pathway, and thereby affecting inflammatory responses.

Recently, a role for the circadian clock has been described as another crucial immunoregulator in allergic asthma pathogenesis. In a study, Teppan et al. compared peripheral blood monocytes obtained from healthy, asthma, and allergic donors. Data showed an altered gene expression in monocytes and macrophages from asthmatic patients compared to healthy controls. These changes in the mononuclear phagocytes due to changes in circadian rhythm may exacerbate chronic inflammatory conditions like asthma. Interference due to these rhythms augmented inflammation and compromised immune resolution by impacting T-cell homeostasis. Regulatory T cells (Tregs) were described to play a critical role in maintaining immune homeostasis by suppressing excessive inflammatory responses caused by unchecked Th2-mediated inflammation. According to Cen et al., the progression of allergic inflammatory responses can be slowed down by increasing FoxP3 expression in Tregs via upregulating the Nrf2 signaling pathway.

Given the complex and multifaceted immune responses involved in allergic asthma, immunologists have stressed the development of targeted therapies to mitigate disease severity. Therapies aimed at regulating Th2 cytokines (IL-4, IL-5, and IL-13) have demonstrated effectiveness at regulating eosinophilia, goblet cell metaplasia, and IgE overexpression in inflamed airways. Furthermore, strategies concentrated on enhancing Treg function or targeting oxidative stress and epigenetic mechanisms are being explored as potential therapeutic approaches. Using an OVA-induced murine model, Cen et al. reported diminished IL-4, IL-5, and IL-13 and increased IL-10 and TGF-β, suggesting the involvement of inflammation in the initiation and progression of allergic asthma. The original research article by Kim et al. highlighted the efficacy of green tea extract (GTE) in regulating inflammatory cellular influx, pro-inflammatory cytokines, and airway hyperresponsiveness (AHR) through inhibiting oxidative stress and the phosphorylation of MAPKs. GTE was shown to have potential as a regulator of allergic asthma response. Like GTE, dimethyl fumarate (DMF) alleviates allergic asthma symptoms by potentiating Nrf-2 signaling in Tregs (Cen et al.). Additionally, the protective role of DEK protein deficiency was associated with suppressed eosinophilia, mitophagy, and NLRP3 inflammasome activation against house dust mite-induced asthma. This study recognized a DEK/ATAD3A/DRP1 signaling axis that regulates mitochondrial dynamics and oxidative damage in asthmatic immune responses (Bai et al.).

Antibodies are biological protein molecules produced by B-lymphocytes naturally or in response to self or foreign molecules called antigens. Some allergens (for example peanut or house dust mite) with antigenic properties induce the production of immunoglobulin molecules (Ig) (2, 3). There are five different classes of immunoglobulins or antibodies: IgA, IgD, IgE, IgG, and IgM. Each of them differs in having a unique heavy chain. Among these antibodies, IgE triggers allergic reactions by binding to the mast cells or basophils, releasing histamines. The role of IgG subclasses is demonstrated to negatively regulate IgE-mediated responses by inducing plasticity in the CD4^+^ T cell populations (4). There is an increase in the global occurrence of IgE-mediated allergy responses, thus, further understanding the mechanism of IgG-mediated regulation of IgE response is critical for developing effective treatments. Furthermore, restricting IgE-FcϵRI-mediated inflammation also offers a potential target for future therapies. Additionally, pharmacological modulation directed at specific ligands, receptors, or signaling pathways involved in the progression of asthma and allergies could be considered for future treatment regimens. The ALKBH5-mediated m6A dysregulation may contribute to airway inflammation by affecting the MAPK pathway, offering potential therapeutic targets for modulating gene expression and inflammatory responses.

The progression of allergic asthma is driven by complex immune mechanisms involving Th2 cells, pro-inflammatory cytokines, IgE class switching, oxidative stress, and epigenetic modifications. Understanding these processes will unlock new opportunities for targeted therapies to modulate allergic responses and improve clinical consequences. In this regard, the development of personalized treatments based on the patient’s unique immunological profile may become a reality, generating anticipation for more effective management of asthma and allergic diseases.
